# The tumor targeting performance of anti-CD166 Probody drug conjugate CX-2009 and its parental derivatives as monitored by ^89^Zr-immuno-PET in xenograft bearing mice

**DOI:** 10.7150/thno.44334

**Published:** 2020-04-27

**Authors:** Marion Chomet, Maxime Schreurs, Margaret Nguyen, Bruce Howng, Ruth Villanueva, Michael Krimm, Olga Vasiljeva, Guus A.M.S van Dongen, Danielle J. Vugts

**Affiliations:** 1 Amsterdam UMC, Vrije Universiteit, Dept. Radiology and Nuclear Medicine, Tracer Center Amsterdam, De Boelelaan 1117, Amsterdam, The Netherlands.; 2 CytomX Therapeutics, Inc., 151 Oyster Point Blvd., Suite 400, South San Francisco, CA 94080, USA.

## Abstract

Probody^®^ therapeutics are recombinant masked monoclonal antibody (mAb) prodrugs that become activated by proteases present in the tumor microenvironment. This makes them attractive for use as Probody drug conjugates (PDCs). CX-2009 is a novel PDC with the toxic drug DM4 coupled to an anti-CD166 Probody therapeutic. CD166 is overexpressed in multiple tumor types and to a lesser extent by healthy tissue.

**Methods**: The tumor targeting potential of CX-2009 was assessed by performing ^89^Zr-immuno-PET/biodistribution/therapy studies in a CD166-positive H292 lung cancer mouse model. Head-to-head comparisons of CX-2009 with the Probody therapeutic without DM4 (CX-191), the unmasked antibody drug conjugate (ADC) CX-1031, and the parental mAb CX-090 were performed. All constructs were ^89^Zr labeled in a GMP compliant way, administered at 10, 110, or 510 µg, and ex vivo biodistribution was assessed at 24, 72, and 168 hours post-injection.

**Results**: Comparable biodistribution was observed for all constructs, confirmed with PET/CT. Tumors showed the highest uptake: 21.8 ± 2.3 ([^89^Zr]Zr-CX-2009), 21.8 ± 5.0 ([^89^Zr]Zr‑CX-191), 18.7 ± 2.5 ([^89^Zr]Zr-CX-1031) and 20.8 ± 0.9 %ID/g ([^89^Zr]Zr-CX-090) at 110 µg injected. Increasing the dose to 510 µg resulted in lower tumor uptake and higher blood levels for all constructs, suggesting receptor saturation. In addition, CX-2009 and CX-1031 showed similar therapeutic potential.

**Conclusions**: CX-2009 is optimally capable of targeting CD166-expressing tumors when compared with its derivatives, implying that enzymatic activation inside the tumor, required to allow CD166 binding, does not limit tumor targeting*.* Because CX-2009 does not bind to mouse CD166, however, reduced targeting of healthy organs should be confirmed in ongoing clinical ^89^Zr-immuno-PET studies.

## Introduction

Antibody drug conjugates (ADCs) are showing a growing clinical utility 1,2 and in recent years the United States Food and Drug Administration (FDA) approved brentuximab vedotin in 2011 (Adcetris^®^, CD30-positive Hodgkin lymphoma and anaplastic large-cell lymphoma), trastuzumab emtansine in 2013 (Kadcyla^®^, HER2NEU 3-positive breast cancer), inotuzumab ozogamicin in 2017 (Besponsa^®^, adults with CD20-positive relapsed or refractory B cell precursor acute lymphoblastic leukemia [ALL]) and gemtuzumab ozogamicin in 2017 (Mylotarg^®^,^,^ newly diagnosed CD33-positive acute myeloid leukemia [AML]). In 2019, polatuzumab vedotin (Polivy^®^, relapsed or refractory diffuse large B cell lymphoma), enfortumab vedotin-ejfv (Padcev^®^, locally advanced or metastatic urothelial carcinoma) and finally fam-trastuzumab deruxtecan-nxki (Enhertu^®^, unresectable or metastatic HER2-positive breast cancer) were approved. Moreover, approximately 80 ADCs are currently being evaluated in clinical trials 1,2.

ADCs consist of a monoclonal antibody (mAb) to which a toxic payload is coupled via a cleavable or non-cleavable linker, preferably without altering the binding and pharmacokinetic properties of the mAb. When reaching its target, the ADC ideally should be internalized, followed by release of the drug intracellularly, with a preserved potency to kill the targeted cancer cells. First-generation ADCs contained classic chemotherapeutic compounds such as doxorubicin as the payload, but these conjugates showed a limited therapeutic efficacy, likely due to the low potency of the payload 3. Second-generation ADCs were therefore equipped with extremely potent payloads. Typically, these payloads are so potent that their narrow therapeutic window prohibits their use as free drugs. The FDA approval of a number of second-generation ADCs (see above) confirms the clinical potential of ADCs. However, despite the growing interest in ADCs and the continuous efforts toward technological improvements (eg, by introduction of more potent drugs and new linker technologies as described in several recent reviews), regulatory approvals of ADCs are stagnating, with several ADCs failing very recently 4,5. One of the lessons learned from clinical ADC development thus far is that many clinical failures are due to unforeseen toxicities. The balance between ADC potency and safety appears to be critical, and efforts to maximize the therapeutic window continue to be crucial 6-11. To redefine the characteristics of an “ideal” ADC, and taking present-day knowledge into account, the initial concept of ADCs might be reconsidered. Essentially, the ADC concept was based on the tumor selectivity of the antibody, resulting in delivery of the drug to targeted tumor cells but not to healthy cells. It is obvious that the characteristics of the target antigen as well as of the antibody are of key importance for the appropriate tumor-selective delivery of ADCs and for avoiding toxic effects in normal tissues 12,13. The suitability of a target antigen depends on its tumor specificity, absolute level and homogeneity of expression, accessibility, and internalization potential. Also, the dose of an ADC and its affinity for the target antigen are expected to be important parameters for enabling homogeneous tumor targeting and effective therapy. Heterogeneous tumor uptake of an ADC might result in “overkill” of a fraction of tumor cells, while other tumor cells remain unaffected. The importance of homogenous tumor targeting was elegantly demonstrated recently by Cilliers et al. In tumor-bearing mice that were treated with Kadcyla^®^, coadministration of unconjugated trastuzumab caused more homogenous tumor uptake as well as concomitant improved anticancer efficacy 14.

**Figure 1:**
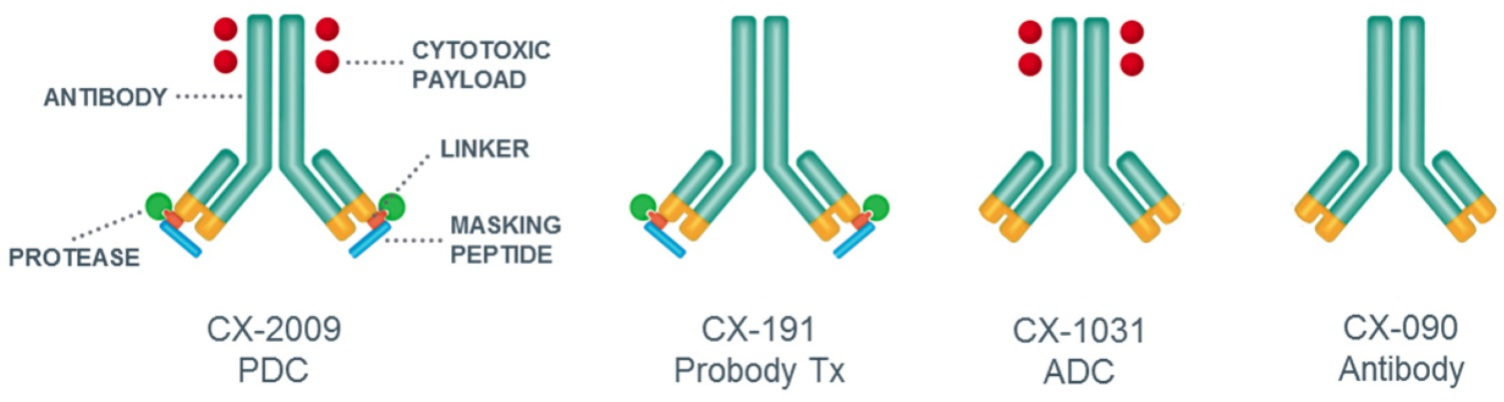
General representation and structure of CX-2009 PDC, CX-191 Probody therapeutic (Probody Tx), CX-1031 ADC, and CX-090 parental antibody.

Unfortunately, only a limited number of tumor antigens have a desirable expression profile for ADC approaches, and therefore many ADCs under clinical development are directed against the same target antigens, with HER2 being the most pronounced example 5,15. Probody therapeutics, under development by CytomX Therapeutics, Inc., represent a potential new approach for improving the selectivity and homogeneity of tumor targeting by antibodies, widening the therapeutic window of ADCs, and increasing the number of candidate target antigens that are suitable for ADC approaches. Probody therapeutics are recombinant antibody prodrugs in which the antigen binding domains are “masked” and then converted to active antigen binding antibodies inside the tumor microenvironment by tumor-associated proteases. As shown in Figure 1, parental mAbs are modified by the recombinant addition of a prodomain, which includes a masking peptide and a protease-cleavable linker, at the amino-terminus of the light chain. The cleavable linker of the prodomain has been optimized to be cleaved by protease(s) that are localized, overexpressed, and extracellularly available in tumor tissues. After activation by proteases and removal of the masking peptide, the mAb is able to bind its target in vivo 16,17.

First proof of concept was obtained with an anti-EGFR Probody construct based on cetuximab. The importance of protease activity in the ability of the anti-EGFR Probody therapeutic to bind its target in vivo has been demonstrated with optical imaging by inhibiting matriptase activity with an anti-matriptase antibody, resulting in a decreased ability of the Probody therapeutic to bind EGFR in vivo 18. Furthermore, when evaluated in nonhuman primates (NHPs), the anti-EGFR Probody therapeutic presented an improved safety profile and increased half-life 19. In these studies, it was demonstrated that the Probody therapeutic remains masked until activated by proteases in the tumor microenvironment. Those encouraging results suggested that Probody therapeutics may also be ideal carriers for selective delivery of drugs to tumors and, thus motivated the development of Probody drug conjugates (PDCs).

Given its high expression in multiple tumor types, CD166, also known as activated leukocyte cell adhesion molecule (ALCAM), appeared to be an attractive target for ADC approaches 20-22. This antigen is overexpressed at the outer cell surface of multiple tumor types such as breast, prostate, lung, or ovarian cancers; however, CD166 is also present in several healthy organs such as colon, stomach, pancreas, thyroid, uterus, and prostate 23-24. Its biological role is still unclear, while internalization of the antigen upon binding to the antigen has been demonstrated 20. The expression pattern of CD166 in tumor and healthy tissues thus makes it an ideal target for the PDC approach. CX-2009 is an anti-CD166 PDC obtained by conjugation of CX-191 anti-CD166 Probody therapeutic via the bifunctional SPDB linker to the maytansinoid drug DM4 (N‑succinimidyl 4-(2-pyridyldithio) butanoate-N2´-deacetyl-N2´-(4-mercapto-4-methyl-1-oxopentyl)-maytansine) 22. SPDB-DM4 was developed by and licensed from ImmunoGen.

The objective of the present study was to evaluate the tumor targeting performance of CX-2009 in vivo using a CD166-positive H292 lung cancer xenograft model. To enable this, CX-2009 was radiolabeled with zirconium-89 (^89^Zr), and ^89^Zr-immuno-Positron emission tomography (^89^Zr-immuno-PET) imaging and biodistribution studies, as initially introduced by our group for antibody and ADC development 25-27, were performed. In this setting, CX-2009 was compared with its benchmark derivatives, as follows: the corresponding Probody therapeutic counterpart without DM4 (CX-191), the unmasked ADC counterpart (CX-1031), and the parental mAb (CX-090) (Figure 1). In addition, CX-2009 was compared with CX-1031 for its therapeutic potential in the same animal model.

## Materials and Methods

### General materials

Starting reagents and solvents were obtained from Sigma-Aldrich^®^ (dimethylsulfoxide (DMSO), L-histidine - pharmaceutical grade, L-histidine.HCl - pharmaceutical grade, Na_2_CO_3_, oxalic acid), Merck Millipore (sucrose, Tween 20^®^ - pharmaceutical grade) or Invitrogen (1M HEPES). ^89^Zr in 1 mol/L oxalic acid was obtained from PerkinElmer (Boston, Massachusetts, USA). Water was distilled and deionized by means of a Milli-Q^®^ water filtration system (MilliporeSigma, Burlington, Massachusetts, USA). p-Isothiocyanatobenzyl desferrioxamine (DFO-Bz-NCS) was purchased from Macrocyclics, Inc. (Plano, Texas, USA). Sumo-tagged human CD166 extracellular domain (ECD) protein was produced at CytomX Therapeutics, Inc. (South San Francisco, California, USA), and His-tagged CD166 ECD from mouse (cat# 5005-M008) was purchased from Sino Biological US Inc. (Wayne, Pennsylvania, USA).

### Antibody constructs

The four antibody constructs, CX-2009, CX-191, CX-1031, and CX-090, were produced by CytomX Therapeutics, Inc. CX-2009 is a Probody construct with, on average, 3.5 DM4 molecules coupled per single Probody molecule. CX-2009 was supplied as a sterile lyophilized cake. After reconstitution with 5 mL water for injection (pharmaceutical grade), each vial contained 25 mg CX-2009 in a solution of 10 mM histidine, 8% sucrose, 0.01% Tween 20 (pH 5.5) (5.3 mg/mL). CX-191 is the Probody version of CX-2009 without DM4 and was supplied at a concentration of 9.4 mg/mL in 25 mM succinic acid, 8% sucrose (pH 5.5 ± 0.2). CX-1031 is the ADC equivalent of CX-2009 containing the parental antibody CX-090 conjugated with, on average, 3.7 DM4 molecules coupled per mAb and was supplied at a concentration of 4.4 mg/mL in 10 mM succinate, 250 mM glycine, 0.5% sucrose, 0.01% Tween-20 (pH 5.5). CX-090 is the parental mAb of CX-2009 (unmasked Probody therapeutic, no drug attached) and was supplied at a concentration of 13.28 mg/mL in phosphate-buffered saline (PBS) (pH 7.2).

### Preparation of ^89^Zr-labeled Probody derivatives: CX-2009

#### [^89^Zr]Zr-CX-2009

Since [^89^Zr]Zr-CX-2009 was planned to be evaluated in clinical trials, procedures for the production of each of the radiolabeled constructs as well as the required quality tests were developed in a Good Manufacturing Practice (GMP) compliant way, as has previously been described by our group for at least 10 other biological molecules 28. In short, five mg of CX-2009 (5.3 mg/mL) were diluted to a 5 mg/mL solution with 0.9% NaCl, adjusted to pH 8.9 to 9.1 by the addition of ±130 µL 0.1 M Na_2_CO_3_ and reacted with 5 equivalents of the bifunctional chelator DFO-Bz-NCS in DMSO (5 mM, 32 µL) at 37°C for 30 min essentially as described by Vosjan et al. 26. At the end of incubation, the reaction mixture was applied on a PD10 column (GE Healthcare Life Sciences), and the product DFO-NCS-CX-2009 (herein designated DFO-CX-2009) was collected in 1 mL of 20 mM L-histidine / 240 mM sucrose / 0.01% Tween 20. Radiolabeling of DFO-CX-2009 (350 µL) with ^89^Zr (120 MBq) was performed for 60 min at room temperature in a 2 mL reaction at pH 7 using 0.5 M HEPES for buffering. After labeling, the reaction mixture was applied on a PD10 column and [^89^Zr]Zr-DFO-CX-2009 (herein designated [^89^Zr]Zr-CX-2009) was collected in 2.5 mL 20 mM L-histidine / 240 mM sucrose / 0.01% Tween 20 (pH 5.4-5.6) .

Preparation of [^89^Zr]Zr-CX-191, [^89^Zr]Zr-CX-1031, and [^89^Zr]Zr-CX-090 can be found in **Supplementary materials**.

### Quality controls

#### Radiochemical purity and conjugate concentration, and integrity

The radiolabeled products were checked for their radiochemical purity by size-exclusion high performance liquid chromatography (SE-HPLC) and spin filter analysis. A JASCO HPLC system was equipped with a Superdex^®^ 200 Increase 10/300 GL (30 cm × 10 mm, 8.6 μm) size exclusion column (GE Healthcare Life Sciences) and a guard column using 0.05 M phosphate buffer / 0.15 M NaCl / 0.01 NaN_3_ (pH 6.7) as mobile phase, with a run time of 40 min at 0.75 mL/min. The radioactivity was monitored with an inline NaI(Tl) radiodetector (Raytest Sockett). The radiolabeled antibody constructs eluted at approximately 15 min, and ^89^Zr/^89^Zr-chelator eluted at approximately 27 min. The radiochemical purity was expressed as the percentage of the area under peak of the radiolabeled product on the radioactive channel. The radiochemical purity was also assessed by spin filter analysis. To this end, 4 µL of product was diluted with 96 µL eluent (5% of DMSO and 95% of 20 mM histidine / 240 mM sucrose buffer / 0.01% Tween 20) and applied on a microcon-30 centrifugal filter unit (Ultracel^®^ YM-30, regenerated cellulose, 30 kDa cut-off, Merck Millipore). The solution was spun down for 7 min at 14,000 rpm (Eppendorf 5430). The filter was washed twice with 100 µL eluent and spun down at 14,000 rpm for 7 min after each wash step. The filtrate contained free ^89^Zr/^89^Zr-DFO, while the radiolabeled mAb was left on the filter. Antibody concentration and integrity were assessed on the same SE-HPLC system described above, using the areas under curve on the ultraviolet (UV) channel at 280 nm. The concentration was determined against a calibration curve of the cold compound.

#### Drug to Probody ratio/drug to antibody ratio (DPR/DAR)

The DPR of [^89^Zr]Zr-CX-2009 and DAR of [^89^Zr]Zr-CX-1031 were determined by HPLC by dividing the area under curve of the PDC/ADC peak at 252 nm by the area under curve of the PDC/ADC peak at 280 nm. A ratio of 0.63 ± 0.10 was determined for the cold CX-2009 and CX-1031, equivalent to an average DPR and DAR of 3.5 and 3.7 DM4 per antibody molecule, respectively. The final radioactive products are required to retain these ratios after modification and radiolabeling.

#### Binding assay of the unlabeled constructs

96-well plates (Nunc MaxiSorp™, Invitrogen™) were coated with 200 ng/well of recombinant CD166 protein (sumo-tagged human CD166 ECD protein was produced at CytomX Therapeutics, Inc.) in 0.05 M carbonate buffer. Plates were washed with 3 × 300 µL Tris-buffered saline (TBS), 0.1% Tween 20 (wash buffer), followed by blocking with TBS + 0.5% casein (block) for 1 h at room temperature. Plates were washed three times and incubated with 80 µL of the indicated concentrations of CX-090 or CX-191 for 1 h at room temperature. Plates were washed and incubated with 80 µL of detection antibody (AffiniPure Anti-human IgG, Jackson ImmunoResearch cat #109-035-088) at 1 to 10,000 dilution for 30 to 45 min at room temperature. Detection was performed by the addition of 3,3′,5,5′-tetramethylbenzidine substrate (1-Step Ultra-TMB, Pierce) followed by an equal volume of 1 M hydrochloric acid. Absorbance at 450 nm was then measured and reported as optical density (OD) 450 nm. Data were analyzed using Prism Graphpad, and apparent equilibrium binding constants (*K*_app_) were determined using nonlinear regression four parameter logistic (4-PL) analysis.

#### Binding assay of the radiolabeled constructs

Immunoreactivity of the four radiolabeled constructs was assessed using a microtiter plate-type of CD166 binding assay with a radioactive read-out. Extracellular domain CD166 antigen (His-sumo-CD166-ECD) was supplied by CytomX Therapeutics, Inc. at a concentration of 0.5 mg/mL in PBS + 4% trehalose (pH 7.2). One day before production of the radiolabeled constructs, CD166 was diluted in a coating buffer (15 mM sodium carbonate / 35 mM sodium bicarbonate / 3 mM sodium azide buffer, pH 9.3 to 9.8) to a concentration of 5 µg/mL and was applied to MaxiSorp™ break-apart wells (100 µL/well, Invitrogen™). After overnight incubation at 4°C, excess CD166 antigen was removed, and the wells were washed three times with PBS (150 µL). Subsequently, the plates were blocked with a solution of 1% BSA/PBS (150 µL) for 45 to 60 min at room temperature while shaking. Finally, the plates were washed three times with a solution of 0.05% Tween 20/PBS (200 µL) before incubation with the radioactive derivatives. Because [^89^Zr]Zr-CX-2009 and [^89^Zr]Zr-CX-191 are masked, a recombinant human protease (matriptase) was used for Probody construct activation prior to incubation in antigen-coated plates.

For activation, 90 μL of either [^89^Zr]Zr-CX-2009 or [^89^Zr]Zr-CX-191 at a concentration of 0.5 mg/mL in 20 mM histidine / 240 mM sucrose / 0.01% Tween 20 was incubated with 10 µL of the matriptase solution (0.4 mg/mL; specific activity >10,000 pmol/min/µg, R&D Systems, Inc., Minneapolis, Minnesota, USA) for 4 h at 25°C in a thermomixer without shaking. A serial dilution of the radiolabeled products in 1% BSA/PBS was made in triplicate, with a concentration range of 4 µg/mL to 62.5 ng/mL. One hundred µL of this solution was added to each coated well and incubated overnight at 4°C while shaking. At the highest dilution, binding was also assessed after addition of 100 µg CX-195 (cold anti-CD166 antibody) as a control for nonspecific binding. After 24 h, supernatants of each of the wells were collected. Next, the wells were washed three times with 0.05% Tween20/PBS (200 µL), and the washing fractions were pooled with the supernatants. Wells and supernatants were counted separately in a gamma counter (Wallac LKB-CompuGamma 1282; Pharmacia). Immunoreactivity of [^89^Zr]Zr-CX-2009, [^89^Zr]Zr-CX-191, [^89^Zr]Zr-CX-1031, and [^89^Zr]Zr-CX-090 was expressed as the percentage of radioactivity bound to the CD166-coated wells compared to the total amount of radioactivity (radiolabeled mAb) added to each well.

### Biodistribution studies

#### Ex vivo biodistribution

All animal experiments were performed in accordance with the NIH Principles of Laboratory Animal Care and with Dutch national law (“Wet op de dierproeven”, Stb 1985, 336).

The biodistribution of [^89^Zr]Zr-CX-2009, [^89^Zr]Zr-CX-191, [^89^Zr]Zr-CX-1031, and [^89^Zr]Zr-CX-090 was evaluated in H292 tumor-bearing mice. After at least 1 week of acclimation, female nu/nu mice (received at 8 weeks old, Envigo, Harlan, 18 to 25g) were injected subcutaneously in both flanks with 5 × 10^6^ H292 human lung cancer cells (American Type Culture Collection [ATCC]). Tumor growth was monitored daily, and tumor volume was assessed with a caliper ((length × width × depth) /2) at least twice a week as soon as tumors became detectable. When tumors reached an average volume of approximately 200 mm^3^, mice were randomized and divided into 14 groups of 5 mice each for injection with 100 to 200 µL of the tracers. Injections were performed under anesthesia with inhalation of 2% to 4% isoflurane/O_2_, intravenously via the retro-orbital plexus, with either [^89^Zr]Zr-CX-2009 (10, 110, or 510 µg); [^89^Zr]Zr-CX-191 (10, 110, or 510 µg); [^89^Zr]Zr-CX-1031 (110 or 510 µg); or [^89^Zr]Zr-CX-090 (10, 110, or 510 µg). At 24 and 48 h postinjection (p.i.), blood samples were taken from all mice in all groups until time of euthanization, and at 72 h p.i., all mice were anesthetized, bled, euthanized, and dissected. Biodistribution of 110 μg [^89^Zr]Zr-CX-2009 was assessed in two additional groups of mice, one at 24 h p.i. and one at 168 h p.i. For the 168 h p.i. group, blood samples were taken at 24, 48, and 72 h p.i. Finally, in one additional group, the animals received a blocking dose of 500 µg CX-090 24 h prior injection of 510 µg [^89^Zr]Zr-CX-2009 to demonstrate specificity of tumor targeting, while blood samples were taken at 24 and 48 h p.i., and the mice were sacrificed at 72 h p.i. All mice were injected with, on average, 0.7 ± 0.1 MBq, except the group sacrificed at 168 h p.i. that received 2.1 ± 0.0 MBq. For all mice, blood, tumors, and organs of interest were collected and weighed, and the amount of radioactivity in each sample was measured by a gamma counter (Wallac LKB-CompuGamma 1282; Pharmacia). Radioactivity uptake was calculated as the percentage of the injected dose per gram of tissue (%ID/g). To facilitate presentation and interpretation of biodistribution data, for the convenience of the reader, results were grouped in various combinations in the figures and tables provided, with some redundancy of data presented in the tables.

#### PET imaging studies

PET imaging was performed on a dedicated small animal nanoScan PET/CT scanner (Mediso Ltd., Hungary). Four mice from each of the groups that received 110 µg of either [^89^Zr]Zr-CX-2009, [^89^Zr]Zr-CX-191, [^89^Zr]Zr-CX-1031, or [^89^Zr]Zr-CX-090 were imaged at 24 and 72 h p.i., with additional imaging at 168 h p.i. for [^89^Zr]Zr-CX-2009. Mice were anesthetized by inhalation of 2% to 4% isoflurane/O_2_ during the entire scanning period (1 h duration per time point). A 5 min computed tomography (CT) scan was acquired prior to each PET scan and was used for attenuation and scatter correction purposes. Reconstruction was performed by three-dimensional reconstruction (Tera-Tomo; Mediso Ltd.) with four iterations and six subsets, resulting in an isotropic 0.4 mm voxel dimension. The scanner was cross-calibrated with the dose calibrator and well counter, enabling accurate measurement of standardized uptake values (SUVs). SUVs were calculated as the ratio of the radioactivity activity concentration (kBq/mL) as measured by the PET scanner within the region of interest (ROI), divided by the decay-corrected amount of injected radiolabeled compound corrected for the weight of the animal. The software Amide (GNU General Public License, Version 2, Made.exe 0.9.2) was used to draw and quantify the ROIs, and VivoQuant was used to capture the images that are displayed.

### Immuno-fluorescence staining

During animal dissection, some healthy organs and halved tumors were collected and flash frozen. Detection of CX-2009, CX-191, CX-1031, and CX-090 in tumor, liver, and kidney tissues of H292 xenograft mice was performed by staining with anti-human IgG (Jackson ImmunoResearch, 609-605-213) labeled with Alexa Fluor 647 on 12 µm thick sections. Evaluation of human CD166 expression was performed with the CX-114 antibody (CytomX Therapeutics, Inc.), which is specific for human CD166, and labeled with Alexa Fluor 488. Prior to staining, slides containing frozen tissue sections were thawed at room temperature, fixed with 10% formalin, and blocked with 3% bovine serum albumin (BSA) PBS-T solution. Samples were stained for 1 hour at room temperature using a 1/100 dilution for anti-human IgG and 5 µg/mL for CX-114. After staining with antibodies, slides were washed three times with PBS and counterstained with DAPI Solution (1 µg/mL Thermo Scientific™, 62248) in PBS for 1 min. Slides were then washed again with PBS to get rid of excess DAPI. Stained slides were cover slipped with ProLong™ Gold Antifade Mountant (Invitrogen™, P36930) and were imaged with the Olympus VS120 system.

### Capillary Electrophoresis Immunoassay

H292 xenograft tumor samples were weighted, and homogenates of the tissues were prepared in Pierce™ IP Lysis Buffer (Thermo Scientific) with added Halt™ Protease Inhibitor Cocktail Kit (Thermo Scientific) using Barocycler (Pressure BioSciences, Inc.). Protein lysates in IP lysis buffer with Halt protease inhibitor/EDTA were analyzed with capillary electrophoresis immunoassay on the Peggy Sue™ system (ProteinSimple). Briefly, protein lysates were normalized to 2mg/mL concentration and mixed with Fluorescent 5× Master Mix (ProteinSimple). Mixture was then denatured and reduced at 95°C for 10 min. Activated and intact CX-2009 and CX-191 were detected using an anti-idiotypic antibody (CytomX Therapeutics, Inc) and anti-rat secondary antibody Fc (Jackson ImmunoResearch Laboratories, Inc.). The reaction was then performed on the Peggy Sue machine (ProteinSimple). The concentration of activated and intact CX-2009 and CX-191 was calculated from the respective standard curves using the Compass software (ProteinSimple). The calculated concentration (ng/mL) was then used to determine the amount (ng) of CX-191 and CX-2009 per gram of tissues.

### Therapy study with CX-2009 and CX-1031

Therapy study with CX-2009 and CX-1031 in H292 bearing mice was conducted at CytomX Therapeutics, Inc. The protocol was reviewed and approved by the Institutional Animal Care and Use Committee (IACUC). For H292 xenograft model establishment, 6 to 8 weeks old female nu/nu (Charles River Strain #088) mice were subcutaneously inoculated in the right hind flank with 5 × 10^6^ H292 cells (American Type Culture Collection [ATCC]) suspended 1:1 with Matrigel in serum-free medium.

Once tumors became measurable, tumor volume and body weight measurements were made twice weekly. Tumor volumes were calculated with the formula ((ab^2^)*0.52), where *a* is the longer and *b* is the shorter of two perpendicular diameters, respectively. Mice were randomized and grouped in tumor size-matched cohorts (n = 8 mice per group) with an average tumor volume of approximately 175 mm^3^. Animals were treated intravenously (5 mg/kg) on day 0 and 7 with the PDC CX-2009, the ADC CX-1031 or the non-binding control ADC Synagis-DM4 (indicated as Ctrl-DM4).

### Statistics

The Grubbs outlier test was used to check and remove outliers, and statistical analysis was performed on the tissue uptake values of the different groups of mice using the Welch's T-test for paired data. For biodistribution data Grubbs test is useful to determine if one value within a group of mice deviates too much (lower or higher) from the mean. Welch t-test is a t-test for small groups which does not assume that the variances are equal between the groups. Both assume normal gaussian distribution of the values. Two-sided significance levels were calculated, and *p* < 0.05 was considered to be statistically significant. All graphs were generated using GraphPad Prism 5.02 software.

## Results

### ^89^Zr-Probody therapeutic derivatives and quality controls

**Figure 2:**
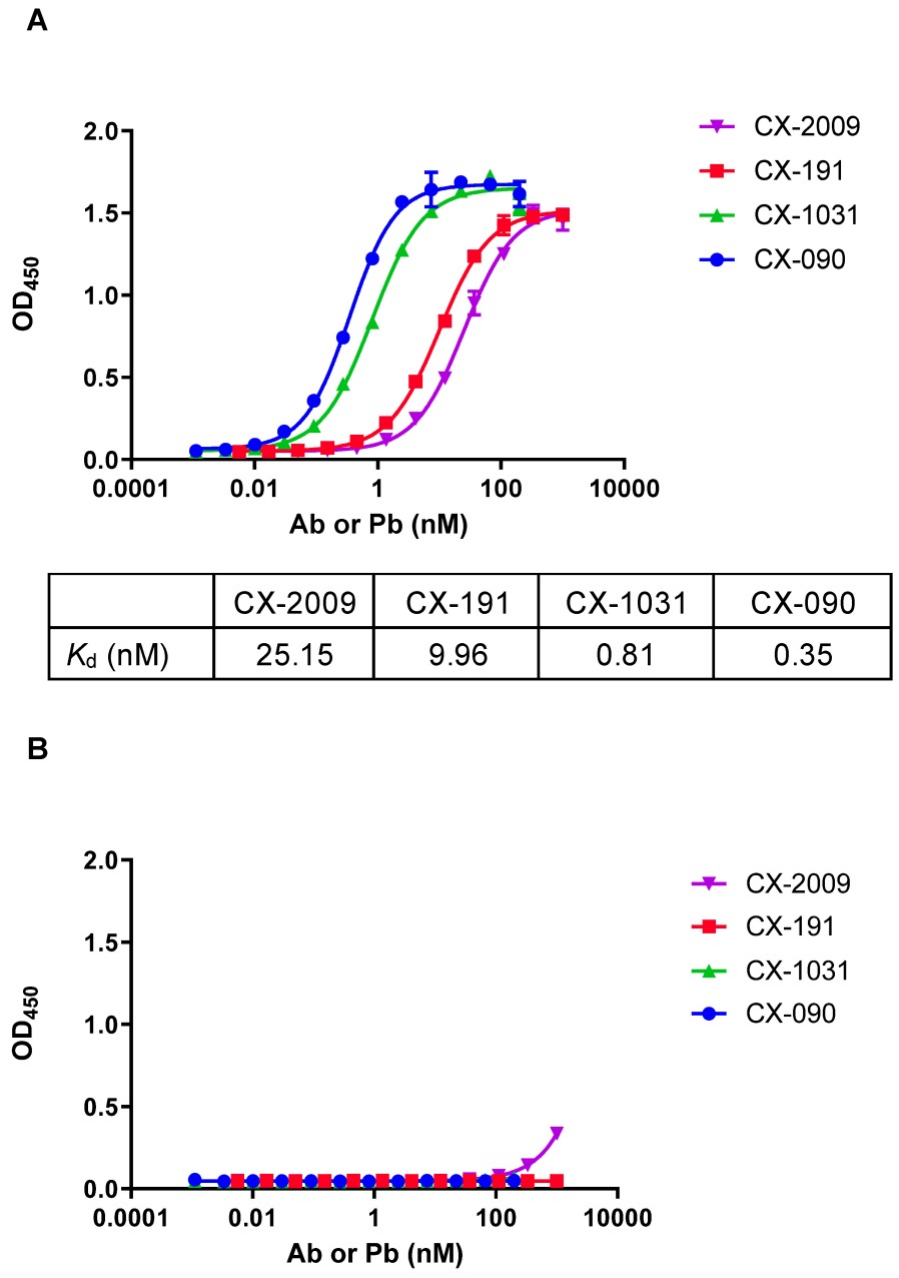
Binding of CX-2009, CX-191, CX-1031, and CX-090 to recombinant CD166 protein from **(A)** human and **(B)** mouse origin. Data points represent the mean of duplicate wells. Error bars show standard error of the mean (SEM). Apparent *K*_d_ (equilibrium dissociation constant) are listed as measured under defined conditions.

The ability of CX-2009, CX-191, CX-1031, and CX-090 to bind recombinant CD166 protein from human or mouse origin was evaluated by direct enzyme-linked immunosorbent assay (ELISA) (Figure 2). Whereas the unmasked ADC counterpart CX-1031 and the parental antibody CX-090 bind with subnanomolar affinity to human CD166 protein with apparent *K*_d_ (*K*_app_) of 0.81 nM and 0.35 nM, respectively **(**Figure 2A), both CX-2009 and CX-191 showed a 29-fold (*K*_app_ = 25.15 nM) and a 31-fold (*K*_app_ = 9.96 nM) decrease in affinity compared to the corresponding CX-1031 (ADC) and CX-090 (mAb), respectively, consistent with masking of antigen binding by the prodomain (Figure 1). No binding to mouse CD166 was detected for any of the four constructs (Figure 2B). After enzymatic activation of CX-2009 and CX-191, their affinities become similar to that of CX-1031 and CX-090, respectively (data not shown).

To enable biodistribution/imaging studies, the constructs were labeled with ^89^Zr. [^89^Zr]Zr-CX-2009, [^89^Zr]Zr-CX-191, and [^89^Zr]Zr-CX-090 were efficiently obtained with radiochemical yields (RCYs) of 62%, 70%, and 81%, respectively. [^89^Zr]Zr-CX-1031 was obtained with a lower RCY of 32%, which was sufficient for the in vivo studies. The radiochemical purities, as assessed by the average of spin filter and high-performance liquid chromatography (HPLC), were above 95% for all constructs. Radiolabeled products showed a similar immunoreactivity, with 74% for [^89^Zr]Zr-CX-2009 and 78% for [^89^Zr]Zr-CX-191 after unmasking with protease, and 82% for [^89^Zr]Zr-CX-1031 and 82% for [^89^Zr]Zr-CX-090. Without prior “unmasking” of the radiolabeled [^89^Zr]Zr-CX-2009 and [^89^Zr]Zr-CX-191 with matriptase, both constructs appeared incapable of binding to CD166 (<5% binding). Binding curves of the six constructs are presented in Figure S1. [^89^Zr]Zr-CX-2009 and [^89^Zr]Zr-CX-1031 presented a ratio of 0.6 ± 0.0 for the peak area at 252/280 nm before and after modification and radiolabeling, indicating that the drug to Probody ratio (DPR) and the drug to antibody ratio (DAR) were preserved, a prerequisite for clinical use of [^89^Zr]Zr-CX-2009.

### Ex vivo biodistribution

**Figure 3:**
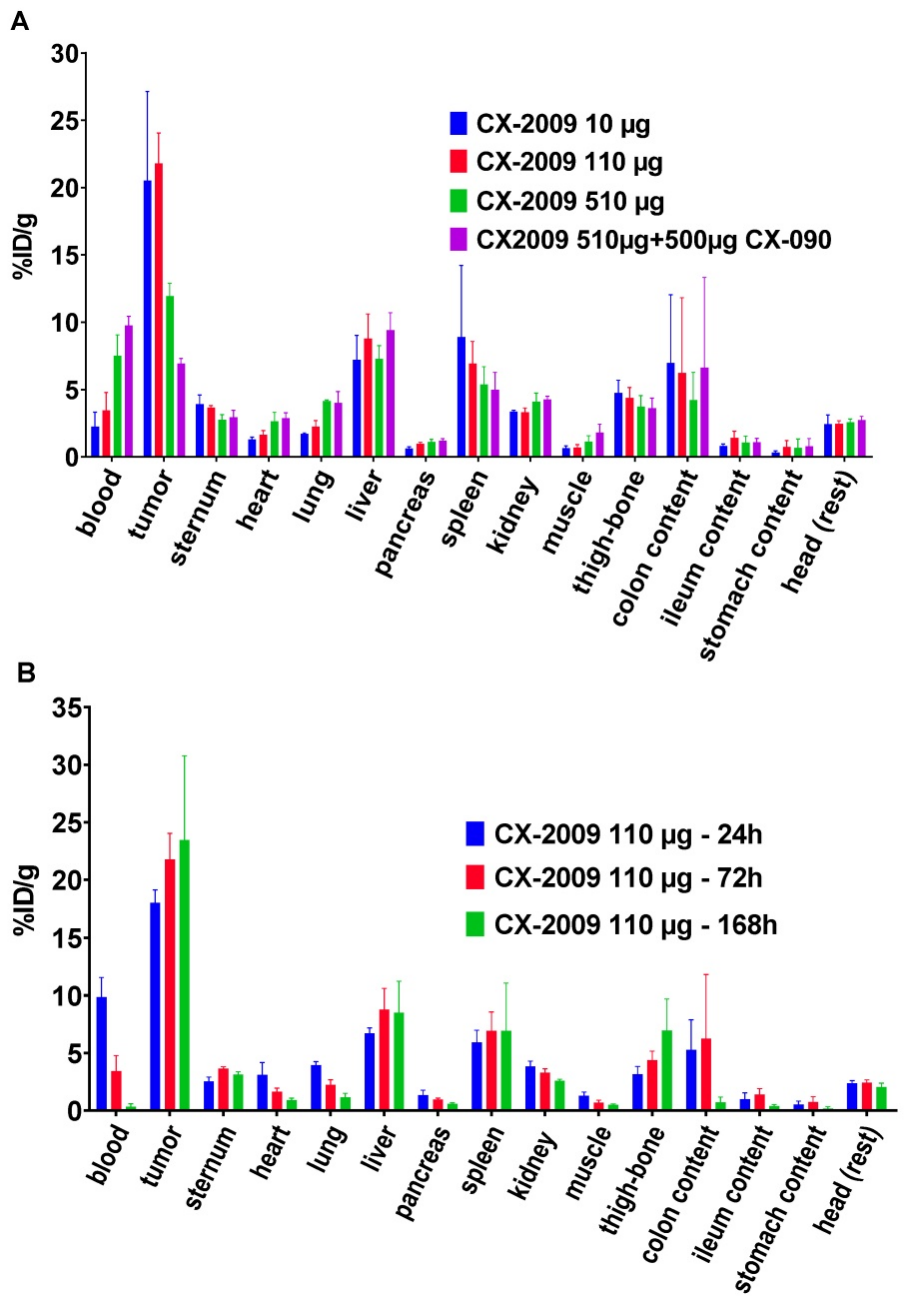
Biodistribution of [^89^Zr]Zr-CX-2009 in H292 tumor-bearing nude mice **(A)** at 72 h p.i. after administration of 10, 110, or 510 µg of the CX-2009 conjugate or 510 µg of the conjugate 24 h post administration of a blocking dose of 500 µg of CX-090 and **(B)** at 24, 72, and 168 h p.i. of 110 µg of the CX-2009 conjugate. Uptake expressed as %ID/g (Mean ±SD, n = 5 animals per group).

For in vivo evaluation of CX-2009 tumor targeting, a CD166-positive H292 xenograft model was chosen, which is characterized by high expression of CD166 (Figure S2). The tracers were intravenously injected 13 ± 1 days after tumor cell administration, when the overall average tumor volume reached 179 ± 108 mm^3^. At the day of sacrifice of the 72-hour postinjection (72 h p.i.) groups, the overall average tumor volume was similar for the different experimental groups, being 264 ± 145 mm^3^. As a first step, the biodistribution of [^89^Zr]Zr-CX-2009 was assessed as a function of dose (10, 110, or 510 µg; Figure 3A and Table S1) and time (24, 72, and 168 h p.i.; Figure 3B and Table S2). Highest [^89^Zr]Zr-CX-2009 tumor uptake of 20.5 ± 6.6 and 21.8 ± 2.3 %ID/g, was observed at 72 h p.i. for the 10 and 110 µg groups, respectively, with lower standard deviations (SD) for the 110 µg group. Tumor uptake values for these two groups were much higher than the blood values of 2.2 ± 1.1 and 3.4 ± 1.3 %ID/g, respectively. Increasing the total dose of [^89^Zr]Zr-CX-2009 to 510 µg resulted in a decreased tumor uptake (11.9 ± 1.0 %ID/g) and increased blood values (7.5 ± 1.6 %ID/g), indicative of target saturation and therefore specific target binding. This hypothesis was supported by administration of an additional 500 µg of the cold parental antibody CX-090 1 day prior to the 510 µg [^89^Zr]Zr-CX-2009 tracer injection, resulting in a further decrease of the tumor uptake to 6.9 ± 0.4 %ID/g (*p* < 0.0001) (Table S1). Based on these results, a dose of 110 µg was selected for subsequent kinetic evaluation of [^89^Zr]Zr-CX-2009 biodistribution at 24, 72, and 168 h p.i. While blood levels steadily decreased over this time period, tumor uptake slightly increased from 18.0 ± 1.2 %ID/g (24 h p.i.) to 21.8 ± 2.3 %ID/g (72 h p.i.) (*p* < 0.05) and 23.5 ± 7.3 %ID/g (168 h p.i.) (Figure 3B, Table S2). The latter increase was not statistically significant due to the large SD at 168 h p.i. Liver as well as spleen uptake did not statistically differ at 24, 72, and 168 h p.i., as follows: 6.7 ± 0.5, 8.8 ± 1.8, and 8.5 ± 2.7 %ID/g, respectively, for liver and 5.9 ± 1.1, 6.9 ± 1.6, and 6.9 ± 4.1 %ID/g, respectively, for spleen. Based on these results, 72 h p.i. was selected for the comparative biodistribution study.

**Figure 4:**
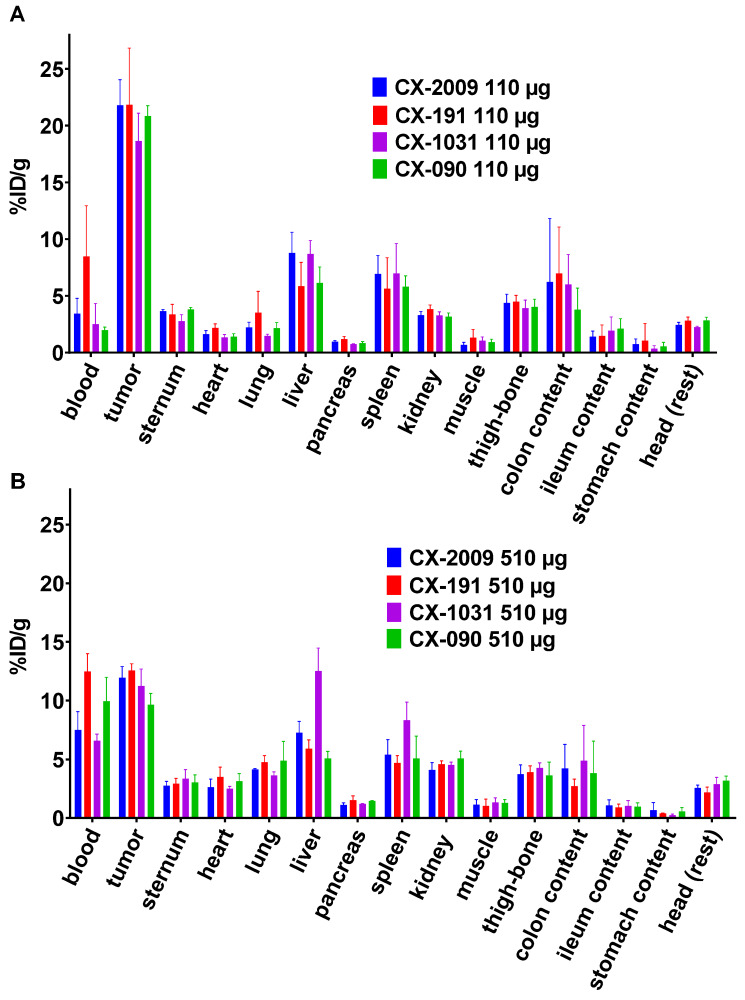
Biodistribution of [^89^Zr]Zr-CX-2009, [^89^Zr]Zr-CX-191, [^89^Zr]Zr-CX-1031, and [^89^Zr]Zr-CX-090 in H292 tumor-bearing nude mice, 72 h after administration of** (A)** 110 µg and **(B)** 510 µg of conjugate. Uptake expressed as %ID/g (Mean ±SD, n = 5 animals per group).

Next, the biodistribution of [^89^Zr]Zr-CX-2009 was compared with the biodistribution of [^89^Zr]Zr-CX-191, [^89^Zr]Zr-CX-1031, or [^89^Zr]Zr-CX-090. A first comparison of biodistribution was performed for the 110 µg groups at 72 h p.i. (Figure 4A, Table S3). [^89^Zr]Zr-CX-2009 presented a tumor uptake of 21.8 ± 2.3 %ID/g, which was not significantly different compared to [^89^Zr]Zr-CX-191 (21.8 ± 5.0 %ID/g), [^89^Zr]Zr-CX-1031 (18.7 ± 2.5 %ID/g), and [^89^Zr]Zr-CX-090 (20.8 ± 0.9 %ID/g). [^89^Zr]Zr-CX-2009 showed a significantly higher liver uptake of 8.8 ± 1.8 %ID/g in comparison with the constructs without DM4 [^89^Zr]Zr-CX-191 and [^89^Zr]Zr-CX-090 (5.9 ± 2.1 and 6.1 ± 1.4 %ID/g, respectively, *p* < 0.05), while no difference was observed with the non-masked ADC [^89^Zr]Zr-CX-1031 (8.7 ± 1.2% ID/g). At this dose, spleen uptake was not different between the four evaluated constructs.

Biodistribution of the constructs was also compared at a dose of 510 µg at 72 h p.i. (Figure 4B, Table S4). At the dose of 510 µg, all tested conjugates showed similar tumor uptake values of 11.9 ± 1.0 ([^89^Zr]Zr-CX-2009), 12.6 ± 0.6 ([^89^Zr]Zr-CX-191), 11.2 ± 1.4 ([^89^Zr]Zr-CX-1031), and 9.7 ± 0.9 %ID/g ([^89^Zr]Zr-CX-090). At this dose, ADC [^89^Zr]Zr-CX-1031 presented a significantly higher liver and spleen uptake in comparison with [^89^Zr]Zr-CX-2009, [^89^Zr]Zr-CX-191, and [^89^Zr]Zr-CX-090, as follows: for liver, 12.5 ± 2.0 %ID/g versus 7.3 ± 1.0, 5.9 ± 0.7, and 5.1 ± 0.6 %ID/g *(p < 0.01)*, respectively; and for spleen, 8.3 ± 1.5 %ID/g versus 5.4 ± 1.3, 4.7 ± 0.6, and 5.1 ± 1.9 %ID/g *(p < 0.05)*, respectively. For unknown reason, [^89^Zr]Zr-CX-191 showed higher blood levels than the other constructs, both after administration of 110 as well as 510 μg (Figure 4 A and B).

**Figure 5:**
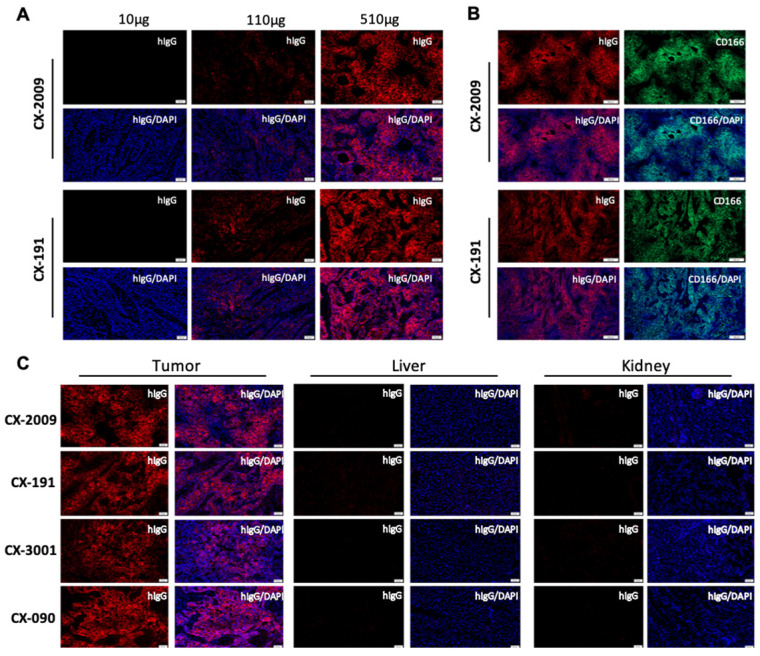
Probody therapeutic preferentially accumulate in the tumor due to target binding. (**A**) Detection of CX-2009 and CX-191 in tumors of H292 xenograft-bearing mice dosed with either [^89^Zr]Zr-CX-2009 or [^89^Zr]Zr-CX-191 at 10, 110, or 510 µg (radiolabeled + cold antibody) and collected at 72 h p.i. (**B**) Similar pattern of CX-2009 and CX-191 intra-tumoral distribution with CD166 expression staining in serial sections of H292 xenografts collected 72 h after dosing of mice with 510 µg CX-2009 or CX-191. (**C**) Immunofluorescence staining of CX-2009, CX-191, CX-1031, and CX-090 in tumor, liver, and kidney of H292 xenograft mice collected 72 h after administration of 510 µg of the respective conjugates. hIgG: staining with anti-human IgG; DAPI: nuclear counter staining with DAPI; CD166: staining for CD166 expression.

**Figure 6:**
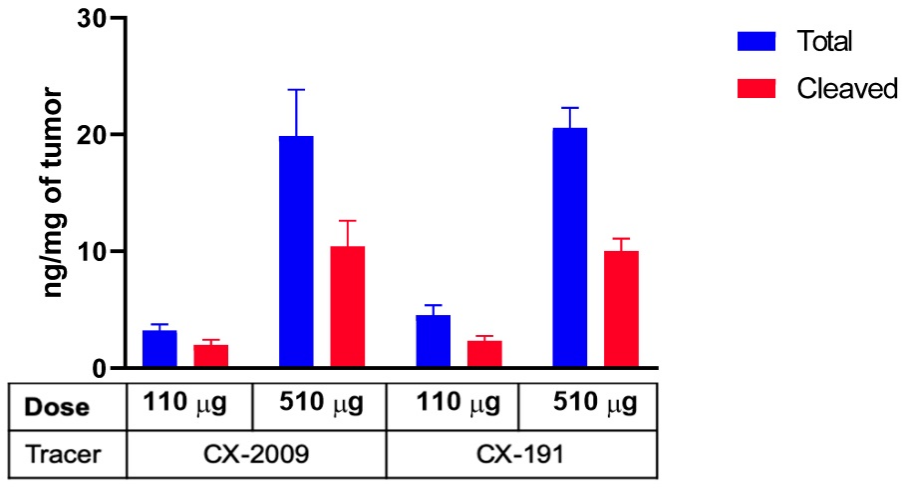
Concentration of total and activated CX-2009 and CX-191 in tumor tissues from H292 xenograft-bearing mice injected with 110 or 510 µg of [^89^Zr]Zr-CX-2009 and [^89^Zr]Zr-CX-191 and collected at 72 h p.i. Concentration expressed as ng/mg of tumor tissue (Mean ±SD, n=5 animals per group for CX-2009: 510 µg and CX-191: 110 µg and 510 µg, n = 4 animals per group for CX-2009: 110 µg).

Distribution of CX-2009 and CX-191 in the tumor tissue was evaluated by immunofluorescence staining with anti-human IgG antibody in the H292 xenograft tumors collected 72 h p.i. of 10, 110, and 510 µg [^89^Zr]Zr-CX-2009 and [^89^Zr]Zr-CX-191, respectively. For both constructs, increases in staining were detected that corresponded with increases in dose (Figure 5A). Furthermore, staining of serial sections of H292 tumors of the 510 μg groups with anti-CD166 antibody noncompeting for the antigen binding with CX-090 and anti-human IgG antibody demonstrated nearly identical staining, thus providing compelling evidence for Probody therapeutic accumulation in the tumor to be driven by the target antigen binding (Figure 5B). Corroborating this hypothesis, no accumulation of CX-2009, CX-191, CX-1031, or CX-090 was detected in normal mouse tissues, such as liver or kidneys that are negative for human CD166 expression, while accumulation in tumors was similar among these constructs (Figure 5C). Furthermore, the concentrations of total and activated CX-2009 and CX-191 constructs were measured in H292-positive tumor tissues collected after administration of 110 and 510 µg, using a capillary electrophoresis immunoassay. As expected based on the [^89^Zr]Zr-CX-2009 and [^89^Zr]Zr-CX-191 tumor uptake data, as assessed by radioactivity measurement, the concentration of total amount of CX-2009 and CX-191 was similar at both evaluated doses: 3.23 ± 1.06 and 4.53 ± 1.94 ng/mg of tissue, respectively, at 110 µg; and 19.86 ± 8.88 and 20.55 ± 3.87 ng/mg of tissue, respectively, at 510 µg (Figure 6.). Notably, no difference in the amount of activated CX-2009 PDC and Probody therapeutic CX-191 was detected at either 110 or 510 µg, as follows: 2.00 ± 0.86 ng/mg versus 2.34 ± 0.96 ng/mg of tumor tissue, respectively, at 110 µg; and 10.42 ± 4.93 ng/mg versus 10.01 ± 2.42 ng/mg of tumor tissue, respectively, at 510 µg. These data thus indicate that conjugation of DM4 to Probody therapeutic CX-191 does not affect intra-tumoral activation of the PDC molecule, resulting in the similar activation rate of both molecules in H292 xenograft tumor microenvironment.

### PET imaging of the 110 µg groups

**Figure 7:**
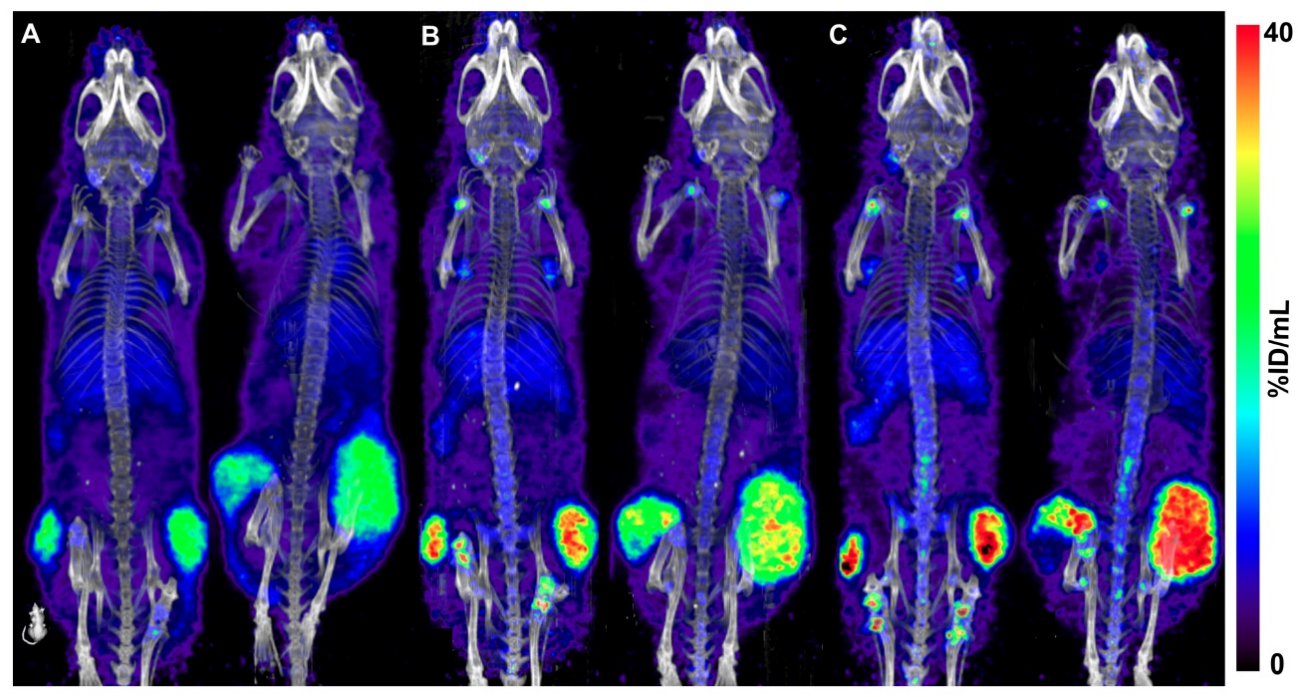
Coronal PET images of two H292 tumor-bearing mice, each injected with 110 µg of [^89^Zr]Zr-CX-2009 and scanned p.i. at 24 h **(A)**, 72 h** (B),** and 168 h **(C)**. Images are decay corrected and are Maximum Intensity Projections (MIP).

Quantitative PET imaging confirmed the similar uptake of the four constructs in the tumors. At 24 h p.i., mean SUVs of 4.3 ± 0.2 for [^89^Zr]Zr-CX-2009, 4.2 ± 0.4 for [^89^Zr]Zr-CX-191, 4.4 ± 0.4 for [^89^Zr]Zr-CX-1031, and 4.4 ± 0.4 for [^89^Zr]Zr-CX-090 were measured for the tumors. At 72 h p.i., SUVs of the tumors remained similar, being 4.8 ± 0.7 for [^89^Zr]Zr-CX-2009, 4.8 ± 0.4 for [^89^Zr]Zr-CX-191, 4.8 ± 0.8 for [^89^Zr]Zr-CX-1031, and 4.9 ± 0.6 for [^89^Zr]Zr-CX-090. Finally, the same mice from the 110 µg [^89^Zr]Zr-CX-2009 group imaged at 168 h p.i. presented a tendency toward a more selective tumor uptake, with a SUV of 5.6 ± 1.9. In Figure 7, representative PET images of [^89^Zr]Zr-CX-2009 obtained at 24 h, 72 h, and 168 h after injection of 110 µg are presented.

### Therapy study with CX-2009 and CX-1031

**Figure 8:**
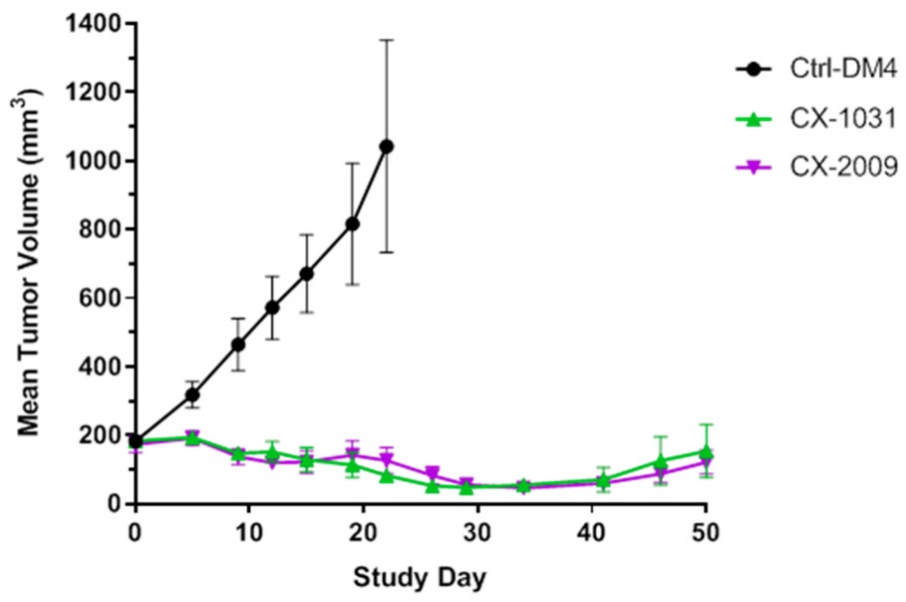
In vivo efficacy in H292 xenograft tumor bearing mouse model treated with 5 mg/kg of non-binding Synagis antibody control conjugated to DM4 (Ctrl-DM4), CX-2009 PDC and corresponding CX-1031 ADC (n = 8 animals per group). Intravenous dosing of compounds was carried out on day 0 and 7. Mean tumor sizes are displayed for each group ± SEM.

In previous experiments it was demonstrated that PDC CX-2009 and ADC CX-1031 show similar targeting of H292 xenografts. To test whether similar tumor uptake results in similar anti-tumor effects, an efficacy study was performed in H292 xenograft mice. As shown by Figure 8 similar efficacy appeared to be the case. While tumor volume rapidly increased from 200 to 1000 mm^3^ within 3 weeks upon treatment with the non-binding control ADC Synagis-DM4, CX-2009 and CX-1031 appeared equally effective and caused reduction of tumor size. Even at 50 days after start of treatment tumor size had not yet recovered to the starting volume of 200 mm^3^. The role of protease activity in tumor accumulation and efficacy of Probody therapeutic in H292 xenograft tumor was additionally confirmed by zymography IHZ^TM^ assay demonstrating *ex vivo* target binding of CX-191, which was abolished by pre-treatment of tumor tissue with protease inhibitors (Figure S3).

## Discussion

In this study, CX-2009, a PDC directed against human CD166, was successfully modified with the chelator DFO-Bz-NCS and radiolabeled with ^89^Zr for immuno-PET molecular imaging studies, ex vivo biodistribution studies and a confirmational therapy study. Radiolabeling was performed in a GMP compliant way to allow the use of [^89^Zr]Zr-CX-2009 as a theranostic in future clinical ^89^Zr-immuno-PET studies. Along with CX-2009, three other constructs were radiolabeled in the same way, namely its corresponding Probody therapeutic counterpart without DM4 (CX-191), its unmasked ADC counterpart (CX-1031), and its parental mAb (CX-090).

To study the tumor targeting performance of CX-2009, biodistribution studies with 10, 110, and 510 µg of [^89^Zr]Zr-CX-2009 were performed in CD166-expressing H292 tumor-bearing mice at 72 h p.i. It is well known from biodistribution studies with conventional antibodies that the level of tumor uptake is dependent on the antibody dose as well as on the expression level of the target antigen in tumor and healthy tissues. The latter is called the “sink effect” and requires dose optimization for every new antibody construct used in humans. In cases of Probody therapeutics such as CX-2009, tumor uptake might also be dependent on tumor associated serine protease and/or matrix metalloproteinase activity. Because CX-2009 is not cross-reactive with mouse CD166, tumor uptake in this case depends on antigen expression in the tumor, antibody dose, and the protease activity that leads to a removal of the masking peptide in the tumor microenvironment. In this study, we have shown that the tumor uptake in the 10 and 110 µg groups was higher than in the 510 µg group: 20.5 ± 6.6 and 21.8 ± 2.3 %ID/g in the 10 and 100 µg groups, respectively, versus 11.9 ± 1.0. %ID/g in the 510 µg group (Figure 3A). Blood levels, however, showed the opposite: 2.2 ± 1.1 and 3.4 ± 1.3 %ID/g for the 10 and 110 µg groups, respectively, versus 7.5 ± 1.6 %ID/g for the 510 µg group. From this experiment it could not be concluded whether the lower tumor uptake in the 510 µg group was due either to antigen saturation or to limited Probody therapeutic activation.

To address both possibilities, the biodistribution of ^89^Zr-CX-2009 was compared with the biodistribution of the three benchmark derivatives, CX-191, CX-1031, and CX-090, in the same animal model. For this purpose, only the 110 and 510 µg dose groups were considered, because the 10 µg dose group had shown biodistribution similar to that of the 110 µg group in the first experiment, although with more variation. These comparative studies revealed three important observations. First, the biodistribution of [^89^Zr]Zr-CX-2009, [^89^Zr]Zr-CX-191, [^89^Zr]Zr-CX-1031, and [^89^Zr]Zr-CX-090 were in general very similar at 110 µg, with tumor uptake levels of 21.8 ± 2.3, 21.8 ± 5.0, 18.7 ± 2.5, and 20.8 ± 0.9 %ID/g , respectively (Figure 4A, Table S3). Second, the biodistribution was also very similar for the four conjugates at 510 µg (Figure 4B, Table S4); however, tumor uptake was reduced for each of the conjugates at 110 µg: 11.9 ± 1.0 for [^89^Zr]Zr-CX-2009, 12.6 ± 0.6 for [^89^Zr]Zr-CX-191, 11.2 ± 1.4 for [^89^Zr]Zr-CX-1031, and 9.7 ± 0.9 %ID/g for [^89^Zr]Zr-CX-090. From these first two observations, it can be concluded that CX-2009 is capable of targeting CD166-expressing tumors when compared with its parental derivatives, indicating that proteolytic activation of CX-2009 inside the tumor, which is required to allow CD166 binding, does not limit tumor targeting, thus supporting the equal therapeutic efficacy of CX-2009 and CX-1031 as demonstrated in the same tumor model (Figure 8). In agreement with this data, the activation dependent target binding of CX-191 Probody construct was demonstrated in H292 tumor tissue using IHZ assay (Figure S3) 29. Furthermore, quantitative analysis of total and unmasked CX-2009 and CX-191 molecule concentrations suggests that drug conjugation did not affect the Probody therapeutic activation rate in H292 tumor tissue (Figure 6). Taken together, these data imply that the reduced tumor uptake at 510 µg is most probably due to antigen saturation. This observation was substantiated when a blocking dose of 500 µg was administered 24 h before injection of 500 µg of the ^89^Zr labeled constructs (Figure 3A). This resulted in further reduction of their tumor uptake, indicating that tumor targeting was CD166 antigen specific. A third observation from these comparative studies is related to normal tissue uptake. While the uptake of the four conjugates in normal tissues was in general quite similar for both dose groups, 110 and 510 µg, this was not the case for all tissues, eg, the liver. [^89^Zr]Zr-CX-2009 (PDC) and [^89^Zr]Zr-CX-1031 (ADC) showed higher liver uptake than did their DM4-free counterparts [^89^Zr]Zr-CX-191 (Probody therapeutic) and [^89^Zr]Zr-CX-090 (antibody) at 110 µg and to a lesser extent at 510 µg for [^89^Zr]Zr-CX-2009. This might indicate that the DPR of 3.5 for [^89^Zr]Zr-CX-2009 and the DAR of 3.7 for [^89^Zr]Zr-CX-1031 may have affected pharmacokinetics and biodistribution compared to the parental mAb. Increased liver uptake with concomitant enhanced blood clearance was recently elegantly demonstrated in ^89^Zr-immuno-PET/biodistribution studies with trastuzumab-auristatin F ADCs, comprising a variable DAR of 0 (trastuzumab without drug), 2.6, or 5.2 11. While the unconjugated antibody and the ADC with a DAR of 2.6 showed very similar biodistribution, indicating inertness of drug coupling, the ADC with a DAR of 5.2 showed fast blood clearance, dramatically increased liver uptake, and strongly reduced tumor uptake.

Antigen-specific targeting of CX-2009 was further confirmed by immunofluorescent staining of tumor, liver, and kidney tissue by analyzing the level and pattern of CD166 expression as well as the level and pattern of CX-2009 tissue binding 72 h after administration to H292 tumor-bearing mice. Also, in these studies the three benchmark constructs were included for comparison. As expected, increased tumor binding of CX-2009 was observed in tumor tissue upon increasing the injected dose from 10 µg to 110 µg and 510 µg (Figure 5A), while no binding was observed in liver and kidney due to lack of antigen expression (Figure 5C). As expected, more intense staining of CX-2009 and CX-191 was detected for the 510 µg group than for the 110 µg group, with homogeneous distribution throughout the tumor (Figure 5A), which was most evidently the case for the 510 µg group. As shown in Figure 5B, the patterns of CX-2009 tumor binding and of CD166 expression looked very similar, indicating that the majority of tumor cells was targeted. As indicated in the introduction section, homogeneous tumor targeting might be an important parameter for ADC efficacy. This aspect will receive more detailed attention in ongoing follow-up studies, including a panel of patient-derived xenograft (PDX) mouse tumor models 30. In addition, in the H292 treatment study CX-2009 has demonstrated tumor reduction similar to its parental ADC version, thus supporting the tumor accumulation data generated with ^89^Zr labeled constructs and, thereby, the PDC concept as tumor targeting ADC pro-drug approach (Figure 8).

While this preclinical proof of concept study demonstrates selective tumor targeting of CX-2009, it cannot address normal tissue protection due to the absence of CX-2009 cross-reactivity with mouse CD166. Nevertheless, reduced healthy tissue uptake can be assumed based on a strongly prolonged serum half-life, as was demonstrated for CX-2009 in NHPs (cynomolgus monkeys) when compared to CX-1031, which indicates reduced target-mediated drug disposition (ie, “sink effect”) for CX-2009 22. This observation is in line with previous reported data in NHP with the anti-EGFR Probody therapeutic 19. Radiolabeling procedures described herein are suitable for Good Manufacturing Practice-compliant conjugate production 28 and are currently applied in a clinical immuno-PET studies with [^89^Zr]Zr-CX-2009* (Eudra CT number 2017-000625-12)*.

## Supplementary Material

Supplementary methods, figures and tables.
