# Supplementary material for: The tumor targeting performance of anti-CD166 Probody drug conjugate CX-2009 and its parental derivatives as monitored by 89Zr-immuno-PET in xenograft bearing mice
Source: Theranostics. 2020 Apr 27;10(13):5815–28. doi: 10.7150/thno.44334 (PMC7255005; doi:10.7150/thno.44334)
Supplement: Supplementary file 1 — Supplementary methods, figures and tables. [file thnov10p5815s1.pdf]

Supplementary Material

The tumor targeting performance of anti-CD166 Probody drug conjugate CX-2009 and its parental derivatives as monitored by ^{89}Zr-immuno-PET in xenograft bearing mice

Authors: Marion Chomet[1], Maxime Schreurs[1], Margaret Nguyen[2], Bruce Howng[2], Ruth Villanueva[2], Michael Krimm[2], Olga Vasiljeva[2]*†, Guus A.M.S van Dongen[1], Danielle J. Vugts[1]*†

Affiliations:

[1]Amsterdam UMC, Vrije Universiteit, Dept. Radiology and Nuclear Medicine, Tracer Center Amsterdam, De Boelelaan 1117, Amsterdam, The Netherlands.[2]Each affiliation should be a separate paragraph.

[2]CytomX Therapeutics, Inc., 151 Oyster Point Blvd., Suite 400, South San Francisco, CA 94080, USA.

*Corresponding Authors: ovasiljeva@cytomx.com; D.Vugts@amsterdamumc.nl

†Contributed equally

Supplementary Methods:

Preparation of ^{89}Zr-labeled Probody therapeutic derivatives: CX-191, CX-1031 and CX-090

[^{89}Zr]Zr-CX-191

[^{89}Zr]Zr-CX-191 was prepared analogously to [^{89}Zr]Zr-CX-2009. Briefly, 2.5 mg of CX-191 (9.4 mg/mL) were diluted to a 5 mg/mL solution with 0.9% NaCl, followed by adjustment of the pH to 8.9-9.1 with 0.1 M Na_2CO_3 and reacted with 3 equivalents of DFO-NCS in DMSO (5 mM, 10 uL) at 37°C for 30 min. Purification of DFO-CX-191, its radiolabeling with ^{89}Zr (85 MBq) in a 2 mL reaction volume, and purification of [^{89}Zr]Zr-CX-191 were the same as described for [^{89}Zr]Zr-CX-2009.

[^{89}Zr]Zr-CX-1031

CX-1031 was first rebuffered with 0.9% NaCl . To this end, two mg of CX-1031 (4.3 mg/mL) were diluted to 0.5 mL with 0.9% NaCl and applied on a PD10 column. The product was collected in 1.5 mL 0.9% NaCl. The pH of this solution was adjusted to 8.9-9.1 with 0.1 M Na_2CO_3 and further reacted with 5 equivalents of DFO-NCS in DMSO (5 mM, 13 uL) at 37°C for 30 min. Purification of DFO-CX-1031, its radiolabeling with ^{89}Zr (50 MBq) in a 2 mL reaction volume, and purification of [^{89}Zr]Zr-CX-1031 were the same as described for [^{89}Zr]Zr-CX-2009.

[^{89}Zr]Zr-CX-090

Before radiolabeling of CX-090, the additional first step of rebuffering was necessary. To this end, 3 mg of CX-090 in PBS (13.28 mg/mL) were diluted to 0.5 mL with 0.9% NaCl and applied on a PD10 column. CX-090 was collected in a 1 mL 0.9% NaCl solution and its concentration

was determined with Nanodrop. The pH of the CX-090 solution (2.1 mg/mL) was adjusted to 8.9-9.1 with 0.1 M Na_2CO_3 and further reacted with 5 equivalents of DFO-NCS in DMSO (5 mM, 13 uL) at 37°C for 30 min. Purification of DFO-CX-090, its radiolabeling with ^{89}Zr (97 MBq) in a 2 mL reaction volume, and purification of [^{89}Zr]Zr-CX-090 were the same as described for [^{89}Zr]Zr-CX-2009.

Immunohistochemistry staining of CD166

H292 xenograft tumor samples were fixed in 10% formalin and sectioned onto glass slides. Tissue slides were deparaffinized and rehydrated to water then incubated in Dako's Dual Peroxidase solution to block endogenous peroxide. Sample sections were then incubated with 2.5% normal goat serum blocking solution. Primary CD166 antibodies (Abcam ab109215, lotGR187770-4) were incubated on tissues section at 5 ug/ml for 60 minutes, washed in TBST then amplified in anti-Rabbit Envision solution (#4003, Dako) for 30 minutes at room temperature. Slides with no primary antibody, serum only, were used as control. 3,3' diaminobenzidine (DAB) substrate was used for chromogenic detection (Dako). Sections were counterstained with hematoxylin, dehydrated and cleared before being coverslipped with Permount mounting media (Fisher Scientific). Slides were imaged with Olympus VS120 slide scanner.

In situ zymography IHZ assay

H292 xenograft tumor samples were flash-frozen in liquid nitrogen and embedded in OCT (optimal cutting temperature) compound. Frozen tissue sections (12 µm) were cut on a cryostat (Microm HM550, ThermoFisher) and placed on glass slides. A solution containing CX-191 (1

μg/ml) was applied on the tissue and incubated for 48 hours at 37°C in 50 mM tris-HCl buffer (pH 7.4), containing 150 mM NaCl, 100 mM $ZnCl_2$, 5 mM $CaCl_2$, and 0.05% Tween 20 in the presence and absence of 1:100 dilution of broad spectrum inhibitor cocktail set III (539134, EMD Millipore, Billerica, MA) and 5 mM EDTA in Tris Buffer (BSPI). The tissue was then washed to remove non-bound material followed by addition of AF647 goat anti-human antibodies (Jackson ImmunoResearch) to label bound CX-191. After washing of unbound secondary antibodies, sections were then counterstained with DAPI and coverslipped using ProLong Gold Anti-Fade Reagent (Invitrogen). Tissue bound CX-191 was visualized with a fluorescence microscope (Olympus IX 81) and an Imaging Software for Life Science Microscopy Cell.

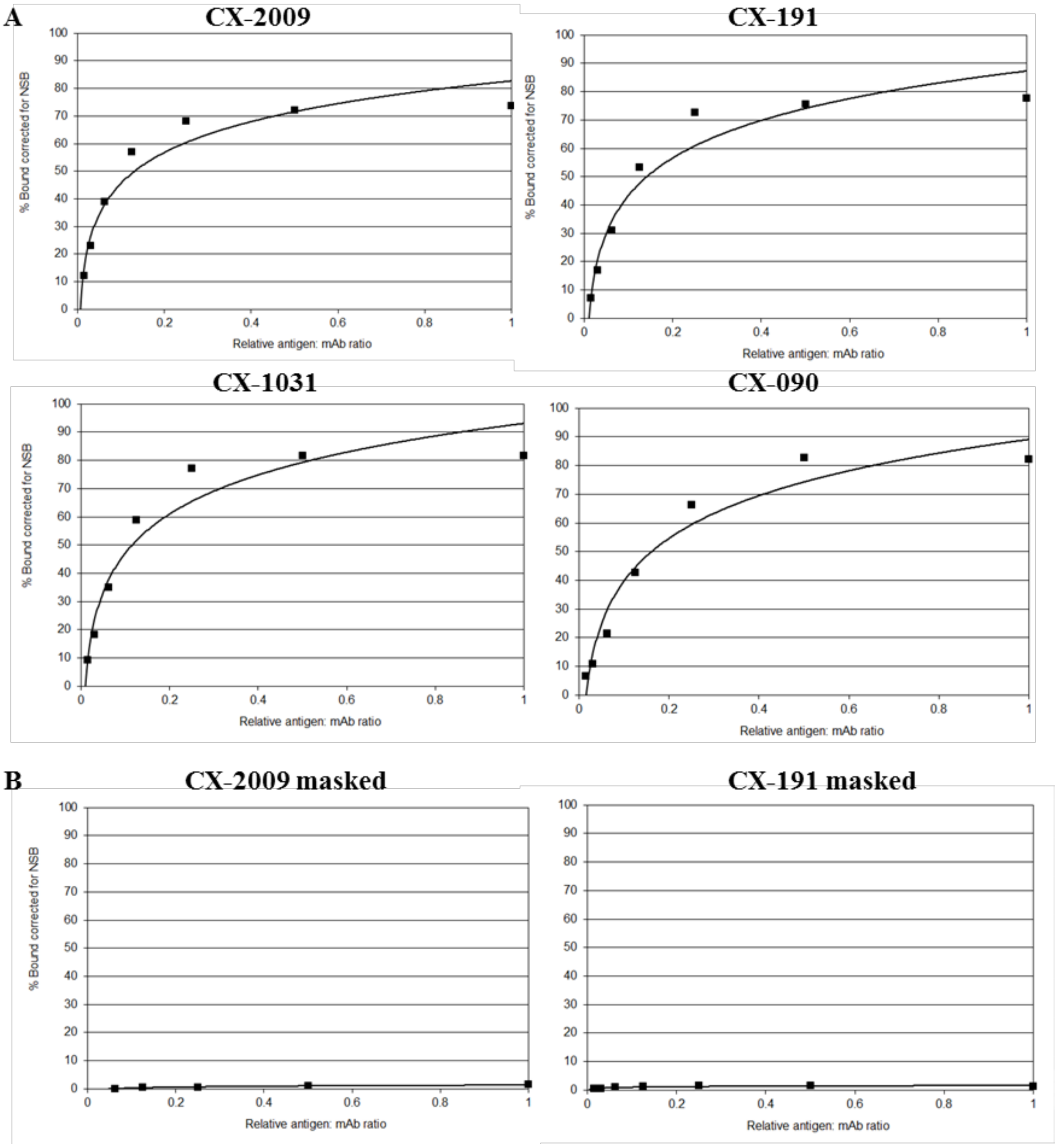

Figure S1. Immunoreactivity binding plots of **(A)** [^{89}Zr]Zr-CX-2009 and [^{89}Zr]Zr-CX-191 after unmasking with recombinant matriptase, and [^{89}Zr]Zr-CX-1031 and [^{89}Zr]Zr-CX-090. In the absence of protease, **(B)** [^{89}Zr]Zr-CX-2009 and [^{89}Zr]Zr-CX-191 present very low immunoreactivity towards CD166.

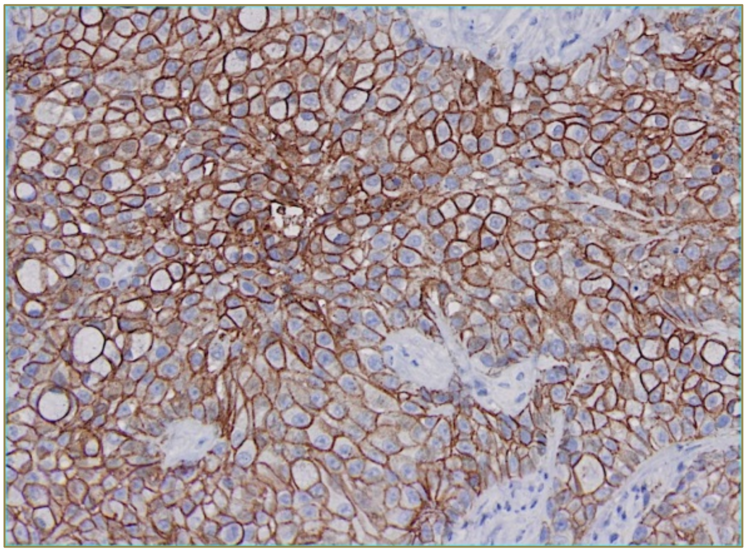

Figure S2. CD166 immunohistochemistry staining of H292 lung cancer xenograft. Scale bar, 100 mm.

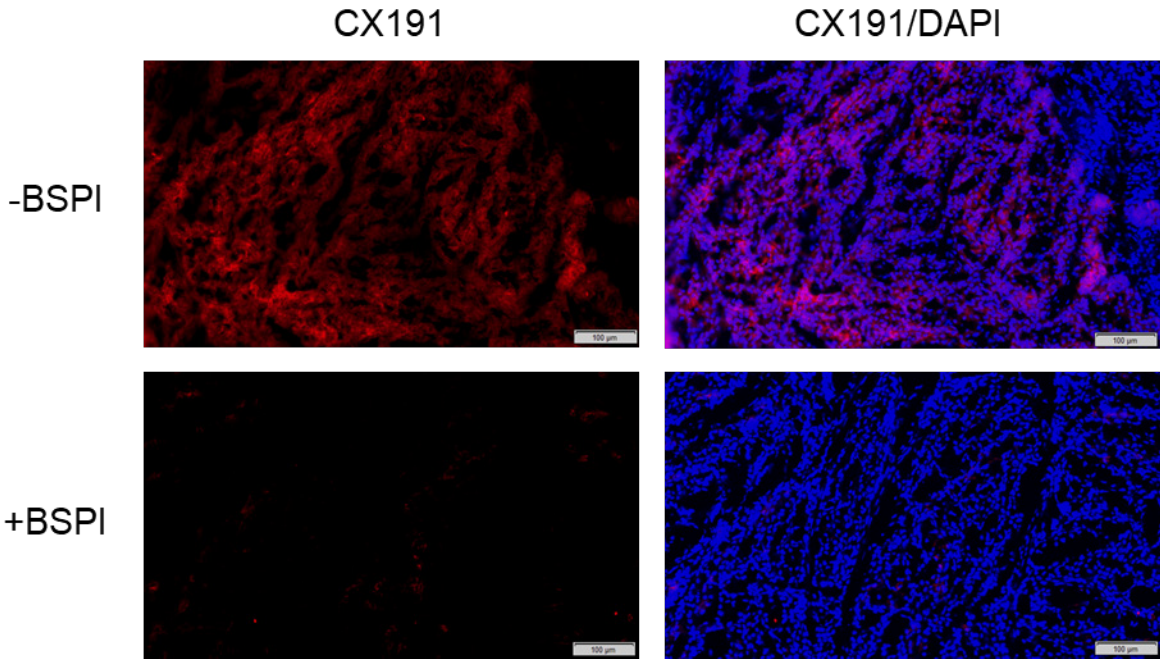

Figure S3. Activation and CD166 target binding of CX-191 in H292 xenograft tumor tissue as determined by the *in situ* zymography IHZ assay. CX-191 Probody therapeutic was incubated with H292 xenograft tumor tissue in the presence and absence of broad-spectrum protease inhibitor (BSPI) cocktail. Red staining indicates the binding of active Probody construct, which is significantly reduced in the presence of BSPI. DAPI nuclear staining appears in blue. Scale bar, 100 μm.

Table S1. Biodistribution of [^{89}Zr]Zr-CX-2009 in H292 tumor-bearing nude mice, 72 h after administration of either 10, 110, 510, or 510 µg of radiolabeled compound in mice injected 24 h prior to tracer injection with 500 µg per mouse of cold CX-090. Results are expressed as mean (%ID/g) ± SD.

	**CX-2009**											
	72 h			72 h			72 h			72 h		
	10 µg			110 µg			510 µg			510 µg + blocking dose of CX-090		
blood	2.2	±	1.1	3.4	±	1.3	7.5	±	1.6	9.8	±	0.7
tumor	20.5	±	6.6	21.8	±	2.3	11.9	±	1.0	6.9	±	0.4
sternum	3.9	±	0.7	3.7	±	0.1	2.8	±	0.4	2.9	±	0.5
heart	1.3	±	0.2	1.6	±	0.3	2.6	±	0.7	2.9	±	0.4
lung	1.7	±	0.1	2.2	±	0.5	4.1	±	0.1	4.0	±	0.8
liver	7.2	±	1.8	8.8	±	1.8	7.3	±	1.0	9.4	±	1.3
pancreas	0.6	±	0.1	1.0	±	0.1	1.1	±	0.2	1.2	±	0.1
spleen	8.9	±	5.3	6.9	±	1.6	5.4	±	1.3	5.0	±	1.3
kidney L	3.4	±	0.1	3.3	±	0.3	4.1	±	0.6	4.3	±	0.2
muscle	0.7	±	0.2	0.7	±	0.2	1.1	±	0.4	1.8	±	0.6
thigh bone	4.8	±	0.9	4.4	±	0.8	3.7	±	0.8	3.6	±	0.8
colon content	7.0	±	5.1	6.2	±	5.6	4.2	±	2.1	6.6	±	6.7
ileum content	0.8	±	0.2	1.4	±	0.5	1.1	±	0.5	1.1	±	0.3
stomach content	0.3	±	0.1	0.7	±	0.5	0.7	±	0.7	0.8	±	0.6
head (rest)	2.4	±	0.7	2.4	±	0.2	2.6	±	0.2	2.7	±	0.3

Table S2. Biodistribution of [^{89}Zr]Zr-CX-2009 in H292 tumor-bearing nude mice, 24, 72, and 168 h after administration of 110 µg of conjugate. Results are expressed as mean (%ID/g) ± SD.

	**CX-2009**								
	24 h			72 h			168 h		
	110 µg			110 µg			110 µg		
blood	9.9	±	1.7	3.4	±	1.3	0.4	±	0.2
tumor	18.0	±	1.2	21.8	±	2.3	23.5	±	7.3
sternum	2.5	±	0.4	3.7	±	0.1	3.1	±	0.2
heart	3.1	±	1.1	1.6	±	0.3	0.9	±	0.2
lung	4.0	±	0.3	2.2	±	0.5	1.2	±	0.3
liver	6.7	±	0.5	8.8	±	1.8	8.5	±	2.7
pancreas	1.3	±	0.4	1.0	±	0.1	0.6	±	0.1
spleen	5.9	±	1.1	6.9	±	1.6	6.9	±	4.1
kidney L	3.8	±	0.5	3.3	±	0.3	2.6	±	0.1
muscle	1.3	±	0.3	0.7	±	0.2	0.5	±	0.1
thigh bone	3.2	±	0.7	4.4	±	0.8	7.0	±	2.7
colon content	5.3	±	2.6	6.2	±	5.6	0.7	±	0.4
ileum content	1.0	±	0.6	1.4	±	0.5	0.4	±	0.1
stomach content	0.5	±	0.3	0.7	±	0.5	0.2	±	0.1
head (rest)	2.4	±	0.2	2.4	±	0.2	2.1	±	0.3

Table S3. Biodistribution of [^{89}Zr]Zr-CX-2009, [^{89}Zr]Zr-CX-191, [^{89}Zr]Zr-CX-1031, and [^{89}Zr]Zr-CX-090 in H292 tumor-bearing nude mice, 72 h after administration of 110 µg of conjugate. Results are expressed as mean (%ID/g) ± SD.

**72 h p.i.**	**CX-2009**			**CX-191**			**CX-1031**			**CX-090**		
	110 µg			110 µg			110 µg			110 µg		
blood	3.4	±	1.3	8.5	±	4.5	2.5	±	1.8	2.0	±	0.3
tumor	21.8	±	2.3	21.8	±	5	18.7	±	2.5	20.8	±	0.9
sternum	3.7	±	0.1	3.4	±	0.9	2.8	±	0.6	3.8	±	0.2
heart	1.6	±	0.3	2.2	±	0.4	1.3	±	0.3	1.4	±	0.3
lung	2.2	±	0.5	3.5	±	1.9	1.5	±	0.2	2.2	±	0.5
liver	8.8	±	1.8	5.9	±	2.1	8.7	±	1.2	6.1	±	1.4
pancreas	1.0	±	0.1	1.2	±	0.3	0.8	±	0.0	0.8	±	0.1
spleen	6.9	±	1.6	5.6	±	2.7	7.0	±	2.6	5.8	±	1.0
kidney L	3.3	±	0.3	3.8	±	0.4	3.3	±	0.3	3.2	±	0.3
muscle	0.7	±	0.2	1.3	±	0.7	1.1	±	0.3	0.9	±	0.3
thigh bone	4.4	±	0.8	4.5	±	0.6	3.9	±	0.7	4.0	±	0.7
colon content	6.2	±	5.6	7.0	±	4.1	6.0	±	2.6	3.8	±	1.9
ileum content	1.4	±	0.5	1.5	±	1.0	1.9	±	1.2	2.1	±	0.9
stomach content	0.7	±	0.5	1.1	±	1.5	0.4	±	0.3	0.6	±	0.4
head (rest)	2.4	±	0.2	2.8	±	0.3	2.3	±	0.1	2.8	±	0.3

Table S4. Biodistribution of [^{89}Zr]Zr-CX-2009, [^{89}Zr]Zr-CX-191, [^{89}Zr]Zr-CX-1031, and [^{89}Zr]Zr-CX-090 in H292 tumor-bearing nude mice, 72 h after administration of 510 µg of conjugate. Results are expressed as mean (%ID/g) ± SD.

**72 h p.i.**	**CX-2009**			**CX-191**			**CX-1031**			**CX-090**		
	510 µg			510 µg			510 µg			510 µg		
blood	7.5	±	1.6	12.5	±	1.5	6.6	±	0.6	9.9	±	2.0
tumor	11.9	±	1.0	12.6	±	0.6	11.2	±	1.4	9.7	±	0.9
sternum	2.8	±	0.4	2.9	±	0.5	3.4	±	0.8	3.0	±	0.7
heart	2.6	±	0.7	3.5	±	0.8	2.5	±	0.2	3.1	±	0.7
lung	4.1	±	0.1	4.8	±	0.6	3.6	±	0.3	4.9	±	1.6
liver	7.3	±	1.0	5.9	±	0.7	12.5	±	2.0	5.1	±	0.6
pancreas	1.1	±	0.2	1.5	±	0.4	1.2	±	0.0	1.4	±	0.1
spleen	5.4	±	1.3	4.7	±	0.6	8.3	±	1.5	5.1	±	1.9
kidney L	4.1	±	0.6	4.6	±	0.3	4.5	±	0.2	5.1	±	0.7
muscle	1.1	±	0.4	1.0	±	0.6	1.3	±	0.4	1.3	±	0.3
thigh bone	3.7	±	0.8	3.9	±	0.6	4.3	±	0.4	3.6	±	1.1
colon content	4.2	±	2.1	2.7	±	0.6	4.9	±	3.0	3.8	±	2.7
ileum content	1.1	±	0.5	0.9	±	0.3	1.0	±	0.5	1.0	±	0.3
stomach content	0.7	±	0.7	0.4	±	0.1	0.2	±	0.1	0.6	±	0.3
head (rest)	2.6	±	0.2	2.2	±	0.5	2.9	±	0.6	3.2	±	0.4
